# Scar architecture affects the electrophysiological characteristics of induced ventricular arrhythmias in hypertrophic cardiomyopathy

**DOI:** 10.1093/europace/euae050

**Published:** 2024-02-20

**Authors:** Pietro Francia, Giulio Falasconi, Diego Penela, Daniel Viveros, José Alderete, Andrea Saglietto, Aldo Francisco Bellido, Julio Martí-Almor, Paula Franco-Ocaña, David Soto-Iglesias, Fatima Zaraket, Dario Turturiello, Antonio Berruezo

**Affiliations:** Arrhythmia Department, Teknon Heart Institute, Teknon Medical Center, C/Vilana 12, 08022 Barcelona, Spain; Cardiology Unit, Department of Clinical and Molecular Medicine, Sant’Andrea Hospital, University Sapienza, Rome, Italy; Arrhythmia Department, Teknon Heart Institute, Teknon Medical Center, C/Vilana 12, 08022 Barcelona, Spain; IRCCS Humanitas Research Hospital, Cardiovascular Department, Milan, Italy; Arrhythmia Department, Teknon Heart Institute, Teknon Medical Center, C/Vilana 12, 08022 Barcelona, Spain; IRCCS Humanitas Research Hospital, Cardiovascular Department, Milan, Italy; Arrhythmia Department, Teknon Heart Institute, Teknon Medical Center, C/Vilana 12, 08022 Barcelona, Spain; Arrhythmia Department, Teknon Heart Institute, Teknon Medical Center, C/Vilana 12, 08022 Barcelona, Spain; Arrhythmia Department, Teknon Heart Institute, Teknon Medical Center, C/Vilana 12, 08022 Barcelona, Spain; Division of Cardiology, Cardiovascular and Thoracic Department, ‘Citta della Salute e della Scienza Hospital, Turin, Italy; Arrhythmia Department, Teknon Heart Institute, Teknon Medical Center, C/Vilana 12, 08022 Barcelona, Spain; Arrhythmia Department, Teknon Heart Institute, Teknon Medical Center, C/Vilana 12, 08022 Barcelona, Spain; Arrhythmia Department, Teknon Heart Institute, Teknon Medical Center, C/Vilana 12, 08022 Barcelona, Spain; Arrhythmia Department, Teknon Heart Institute, Teknon Medical Center, C/Vilana 12, 08022 Barcelona, Spain; Arrhythmia Department, Teknon Heart Institute, Teknon Medical Center, C/Vilana 12, 08022 Barcelona, Spain; Arrhythmia Department, Teknon Heart Institute, Teknon Medical Center, C/Vilana 12, 08022 Barcelona, Spain; Arrhythmia Department, Teknon Heart Institute, Teknon Medical Center, C/Vilana 12, 08022 Barcelona, Spain

**Keywords:** Hypertrophic cardiomyopathy, Magnetic resonance, Scar, Sudden death, Ventricular tachycardia, Programmed ventricular stimulation

## Abstract

**Aims:**

Late gadolinium enhancement cardiac magnetic resonance (LGE-CMR) detects myocardial scarring, a risk factor for ventricular arrhythmias (VAs) in hypertrophic cardiomyopathy (HCM). The LGE-CMR distinguishes core, borderzone (BZ) fibrosis, and BZ channels, crucial components of re-entry circuits. We studied how scar architecture affects inducibility and electrophysiological traits of VA in HCM.

**Methods and results:**

We correlated scar composition with programmed ventricular stimulation-inducible VA features using LGE intensity maps. Thirty consecutive patients were enrolled. Thirteen (43%) were non-inducible, 6 (20%) had inducible non-sustained, and 11 (37%) had inducible sustained mono (MMVT)- or polymorphic VT/VF (PVT/VF). Of 17 induced VA, 13 (76%) were MMVT that either ended spontaneously, persisted as sustained monomorphic, or degenerated into PVT/VF. Twenty-seven patients (90%) had LGE. Of these, 17 (57%) had non-sustained or sustained inducible VA. Scar mass significantly increased (*P* = 0.002) from non-inducible to inducible non-sustained and sustained VA patients in both the BZ and core components. Borderzone channels were found in 23%, 67%, and 91% of non-inducible, inducible non-sustained, and inducible sustained VA patients (*P* = 0.003). All 13 patients induced with MMVT or monomorphic-initiated PVT/VF had LGE. The origin of 10/13 of these VTs matched scar location, with 8/10 of these LGE regions showing BZ channels. During follow-up (20 months, interquartile range: 7–37), one patient with BZ channels and inducible PVT had an ICD shock for VF.

**Conclusion:**

Scar architecture determines inducibility and electrophysiological traits of VA in HCM. Larger studies should explore the role of complex LGE patterns in refining risk assessment in HCM patients.

## Graphical Abstract

**Graphical Abstract:**
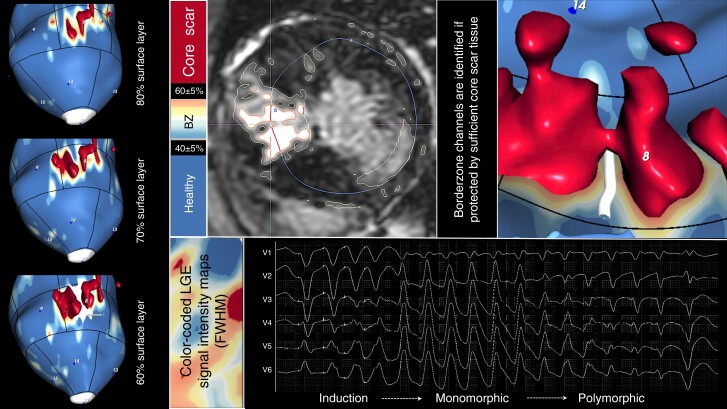
Late gadolinium enhancement cardiac magnetic resonance (LGE-CMR) images were post-processed by creating nine concentric surface layers from endocardium to epicardium of the left ventricular wall thickness, obtaining a 3D shell for each layer. Colour-coded pixel signal intensity (PSI) maps based on LGE-CMR images were projected to each shell. Hyper-enhanced areas were identified as core zone, borderzone (BZ), or healthy tissue using 40 ± 5% and 60 ± 5% of the maximum PSI as thresholds. Borderzone channels were identified as continuous corridors of BZ surrounded by unexcitable scar core. Most programmed ventricular stimulation-induced monomorphic or monomorphic-initiated polymorphic VT/VF had an ECG origin that aligned with the location of LGE and BZ channels.

**What’s new?**

- Late gadolinium enhancement (LGE) extent and borderzone channel mass is increased in hypertrophic cardiomyopathy patients with inducible ventricular arrhythmias.
- Polymorphic VTs/VF induced with programmed ventricular stimulation are often preceded by a short sequence of monomorphic VT.
- Most induced monomorphic VTs or monomorphic-initiated polymorphic VT/VF had an ECG origin that aligns with the location of LGE and BZ channels.

## Introduction

Hypertrophic cardiomyopathy (HCM) is a genetic disease characterized by myocardial hypertrophy, disarray, and fibrosis, leading to increased risk of ventricular arrhythmias (VAs) and sudden cardiac death (SCD).^1,2^ Late gadolinium enhancement cardiac magnetic resonance (LGE-CMR) detects myocardial scarring, a risk factor for VA in HCM.^3–5^ Indeed, extensive LGE has been incorporated as a major risk factor for SCD in current guidelines.^6,7^ Late gadolinium enhancement cardiac magnetic resonance post-processing enables the identification, within the scar, of dense (core), diffuse (borderzone; BZ) fibrosis, and channels of BZ tissue (BZ channels) that connect areas of normal myocardium within unexcitable core zones.^8^ Borderzone channels extending into non-conductive scar tissue can serve as slow-conducting re-entrant pathways and are critical to entail VA in ischaemic and non-ischaemic heart disease.^8–11^ Indeed, in high-risk HCM patients implanted with an ICD, BZ channels predict the occurrence of appropriate ICD interventions for ventricular tachycardia or fibrillation.^12^ However, their pathophysiological role as a substrate of VA in HCM is yet to be defined.

Programmed ventricular stimulation (PVS), which has been largely abandoned in contemporary HCM SCD risk stratification, has been recently reappraised and shown to predict SCD.^13^ Interestingly enough, in HCM patients, the amount of myocardial fibrosis as assessed by LGE is correlated with the inducibility of VA at PVS.^14^

In a series of consecutive patients with HCM who underwent PVS to assist in defining ICD candidacy, we investigated whether myocardial scar architecture, as assessed by CMR, affects the inducibility of VA and establishes substrates that determine electrophysiological characteristics of VA.

## Methods

### Study subjects

We retrospectively assessed 50 patients with HCM who were referred to our institution between November 2018 and May 2023 for risk assessment. The diagnosis of HCM was based on echocardiographic demonstration of a hypertrophied and non-dilated left ventricle in the absence of any other cardiac or systemic disease that could produce a comparable LV hypertrophy.^6,15^ Patients with LV hypertrophy secondary to metabolic/infiltrative diseases were excluded. LV outflow tract obstruction was diagnosed when the peak instantaneous outflow gradient estimated by continuous-wave Doppler was ≥30 mmHg under basal conditions. End-stage HCM was defined as the presence of LV ejection fraction < 50%.

Risk stratification of SCD was conducted according to the ESC 5-year SCD risk score^15^ and the presence of one or more established risk factors for SCD as per AHA guidelines,^6^ including maximal LV thickness ≥ 30 mm, family history of SCD in at least one first-degree relative < 50 years of age, non-sustained ventricular tachycardia (NSVT), recent (≤6 months) unexplained syncope, end-stage disease, apical aneurysm, and extensive LV LGE at CMR.

Programmed ventricular stimulation was considered for all patients to refine risk evaluation. In six high-risk patients, shared decision-making resulted in primary prevention ICD implantation based on clinical risk factors without using PVS. Fourteen patients (7 men; mean age: 61 ± 9 years) with low risk of SCD refused PVS. The remaining 30 patients accepted PVS irrespective of the estimated risk based on ESC/AHA models, forming the study cohort. All patients underwent LGE-CMR. Informed consent for the electrophysiological testing protocol was obtained from all subjects. The study was conducted in accordance with the customary clinical practice at our Institute and was approved by the local ethical committee.

### Late gadolinium enhancement cardiac magnetic resonance post-processing and scar characterization

All LGE-CMR images were analysed according to a previously described protocol.^16^ Briefly, a full LV volume was reconstructed in the axial orientation, and the resulting images were processed with ADAS 3D LV software (Galgo Medical, Barcelona, Spain). Nine concentric surface layers (from 10–90%) were created automatically from endocardium to epicardium of the LV wall thickness, obtaining a 3D shell for each layer. Colour-coded pixel signal intensity (PSI) maps based on LGE-CMR images were projected to each shell, following a trilinear interpolation algorithm. A PSI-based algorithm was applied to characterize hyper-enhanced areas as core zone, BZ, or healthy tissue using 40% ± 5% and 60% ± 5% of the maximum PSI as thresholds, as previously described.^16,17^

The total scar mass, BZ mass, and core mass in each shell were automatically measured using the ADAS 3D LV software. Borderzone channels, defined as continuous corridors of BZ surrounded by unexcitable scar core or an anatomical barrier (e.g. mitral annulus) connecting two areas of healthy tissue, were also automatically identified. The BZ channel mass, defined as the grams of BZ tissue that make up the channel, was obtained by multiplying the number of image voxels within the identified channel by the voxel volume and a myocardial density of 1.05 g/cm^3^, using a full-automated tool embedded within the ADAS 3D LV software.

### Programmed ventricular stimulation

Antiarrhythmic drugs were discontinued before electrophysiological study. One quadripolar catheter was introduced percutaneously through the right femoral vein and positioned at the right ventricular apex under fluoroscopic guidance. Programmed ventricular stimulation was performed using an EP-Tracer stimulator (CardioTek BV, Sittard, The Netherlands). Pulses of 1.0 ms in duration were applied at twice the amplitude threshold.

Programmed ventricular stimulation was performed with up to three extrastimuli at a single three-driving cycle length (600, 500, and 430 ms) from the right ventricular apex until either ventricular refractoriness or a coupling interval of 200 ms for all extrastimuli. The PVS protocol did not involve the use of isoproterenol. Non-sustained VT in response to PVS was considered as a VT of at least 3 beats at heart rate (HR) > 120 bpm. Induced VTs were considered sustained when lasting >30 s or required cardioversion for haemodynamic collapse. A VT was considered monomorphic (MMVT) when the ECG showed similar axis and beat-to-beat morphology over all 12 leads, and polymorphic (PVT) in case of beat-to-beat changes. Ventricular fibrillation (VF) was defined as rapid-rate chaotic asynchronous fractionated ECG activity.

The anatomical localization of LV scar by the AHA 17 segments model was assessed for matching with the predicted site of origin of MMVTs according to a previously described algorithm.^18^ Briefly, the 17-segment model is represented over the QRS axis and limb leads. The process involves two steps. First, the limb lead with the highest voltage magnitude, whether it is positive or negative, is identified. If this magnitude corresponds to leads I, II, or III, an analysis of the adjacent leads is required. The adjacent lead with the higher magnitude will help determine the group of segments that are suggested as a potential site of origin. Secondly, the positivity or negativity of the precordial leads V3 and V4 is identified. If there is positive or negative concordance between the two leads, it indicates that the arrhythmia originates from either a basal or apical location, respectively. Other combinations suggest that the VT originates from a medial location.

### Follow-up

Follow-up was from the date of PVS to the last routine clinical assessment, ICD interrogation or death. Device interrogation reports were collected at each patient evaluation (every 3–6 months) or via home-monitoring to assess whether any appropriate or inappropriate ICD therapies occurred.

ICD interventions were considered appropriate when delivered for VT or VF, and inappropriate in case of supraventricular arrhythmia, noise, or T-wave oversensing.

### Statistical analysis

Continuous variables are given as mean ± standard deviation for normally distributed data or median (interquartile range, IQR) in case of skewed distribution. Categorical variables are given as absolute numbers and percentages. To compare the means of two variables, the Student’s *t*-test or Mann–Whitney test was used, as appropriate. When comparing >2 groups, one-way ANOVA or Kruskal–Wallis test was used, as appropriate. Patients without LGE were also included in the analysis of LGE mass. Proportions were compared using the Fisher’s exact test. All tests were two-sided, and a *P* value of <0.05 was considered statistically significant. Statistical analysis was performed using IBM SPSS Statistics, version 27.0 (IBM Corp; Armonk, NY, USA).

## Results

Thirty consecutive HCM patients (25 males; mean age: 57 ± 12 years) with available CMR underwent PVS to refine risk assessment of SCD and composed the study population. Major risk factors for SCD were unexplained syncope (*n* = 4; 13.3%), family history of SD (*n* = 8; 26.7%), massive LV hypertrophy (*n* = 1; 3.3%), and NSVT (*n* = 9; 30%). Six patients had LV outflow tract obstruction (mean gradient 65 ± 27 mmHg). One patient had LV systolic dysfunction.

According to the risk stratification model endorsed by the European Society of Cardiology,^7^ 2 (6.7%) patients were classified at high, 7 (23.3%) at intermediate, and 21 (70%) at low risk of SD. Of these latter, 10 (33.3%) had a Class IIb indication for a primary prevention ICD because of significant LGE at CMR. The AHA/ACC guidelines^6^ would have indicated an ICD with a Class IIa indication in 16 (53.3%) patients, Class IIb in 7 (23.3%) patients, and no ICD indication in 7 (23.3%).

### Programmed ventricular stimulation

At PVS, 13 patients (43%) had no inducible VA, 6 (20%) had inducible NSVT, and 11 (37%) had inducible mono- or polymorphic sustained VA. Of the six NSVTs, four were induced using three extrastimuli, and two were induced using two extrastimuli. The median duration of induced NSVTs was 2.0 s (IQR: 0.8–3.0). The majority of sustained VA (8 out of 11) was induced with three extrastimuli, while the rest were induced using two. Patients with inducible sustained VA showed a trend towards a higher risk profile (*Table 1*).

**Table 1:** Clinical characteristics of patients according to inducibility of ventricular arrhythmias

Characteristics	Non-inducible or inducible with NSVT (*n* = 19)	Inducible with sustained VT/VF (*n* = 11)	*P*
Male sex, *n* (%)	15 (78.9)	10 (90.9)	0.39
Age (years)	58 ± 10	56 ± 14	0.72
Unexplained syncope, *n* (%)	1 (5.3)	3 (27.3)	0.08
Family history of SD, *n* (%)	3 (15.8)	5 (45.5)	0.07
Max wall thickness (mm)	20 ± 4	21 ± 5	0.56
Max wall thickness > 30 mm, *n* (%)	0	1 (9.1)	0.18
NSVT, *n* (%)	6 (50)	1 (12)	0.21
Left atrium AP diameter (mm)	41 ± 5	39 ± 6	0.23
End-stage, *n* (%)	1 (5.3)	1 (9.1)	0.68
ESC 5-year risk of SD (%)	2.9 ± 1.5	3.0 ± 1.8	0.86
Beta-blockers, *n* (%)	10 (52.6)	8 (72.7)	0.27
Amiodarone, *n* (%)	0	2 (18.2)	0.06

AP, antero-posterior; NSVT, non-sustained ventricular tachycardia; SD, sudden death.

Among non-sustained arrhythmias, three were MMVTs (mean cycle length: 215 ± 26 ms), two were PVT that initiated as monomorphic (monomorphic-initiated PVT, MI-PVT), and one was induced as PVT primarily. Among sustained VAs, three were MMVTs (mean cycle length: 242 ± 51), five were MI-PVT, and three were induced as PVT and degenerated into VF. Therefore, out of 17 induced VA, 13 (76.4%) were MMVT that either ended spontaneously, persisted as sustained monomorphic, or degenerated into PVT/VF. Monomorphic-initiated PVTs were triggered by a short (median: 5 beats, range: 4–6) and rapid-rate (cycle length: 236±45 ms) run of MMVT. The mean cycle length of MMVT that degenerated into PVT was comparable to that of MMVT that either ended spontaneously or persisted as monomorphic (236 ± 45 vs. 228 ± 40 ms, *P* = 0.73).

### Three-dimensional structure of late gadolinium enhancement and borderzone channels

Late gadolinium enhancement was observed in 27 out of 30 patients (90.0%), including 4 patients with small scar mass (<5% of LV mass). Overall, median scar mass was 12.6 g (IQR: 3.8–32.4), representing 9.9% (IQR: 4.8–21.4) of LV mass. The scar was mainly composed of BZ tissue (8.6% of LV mass, IQR: 4.1–18.6), while dense scar represented on average 1.4% of LV mass (IQR: 0.3–2.3).

Borderzone channels were found in 17 (56.6%) out of 30 patients. The median mass of BZ channels was 0.91 g (IQR: 0.59–1.87), with a median length of 22.2 mm (IQR: 16.6–34.1).

### Cardiac magnetic resonance substrate location and VT site of origin

The median time from LGE-CMR to PVS was 1 month (IQR: 0.4–3.0). Patients with and without LGE exhibited distinct responses to PVS. Out of seven patients with minimal (<5%) or no LGE, only one had an inducible NSVT and none had inducible sustained VA. On the contrary, 16 (69.5%) out of 23 patients with ≥5% LGE had either a non-sustained or a sustained inducible VA.

**Figure 1:**
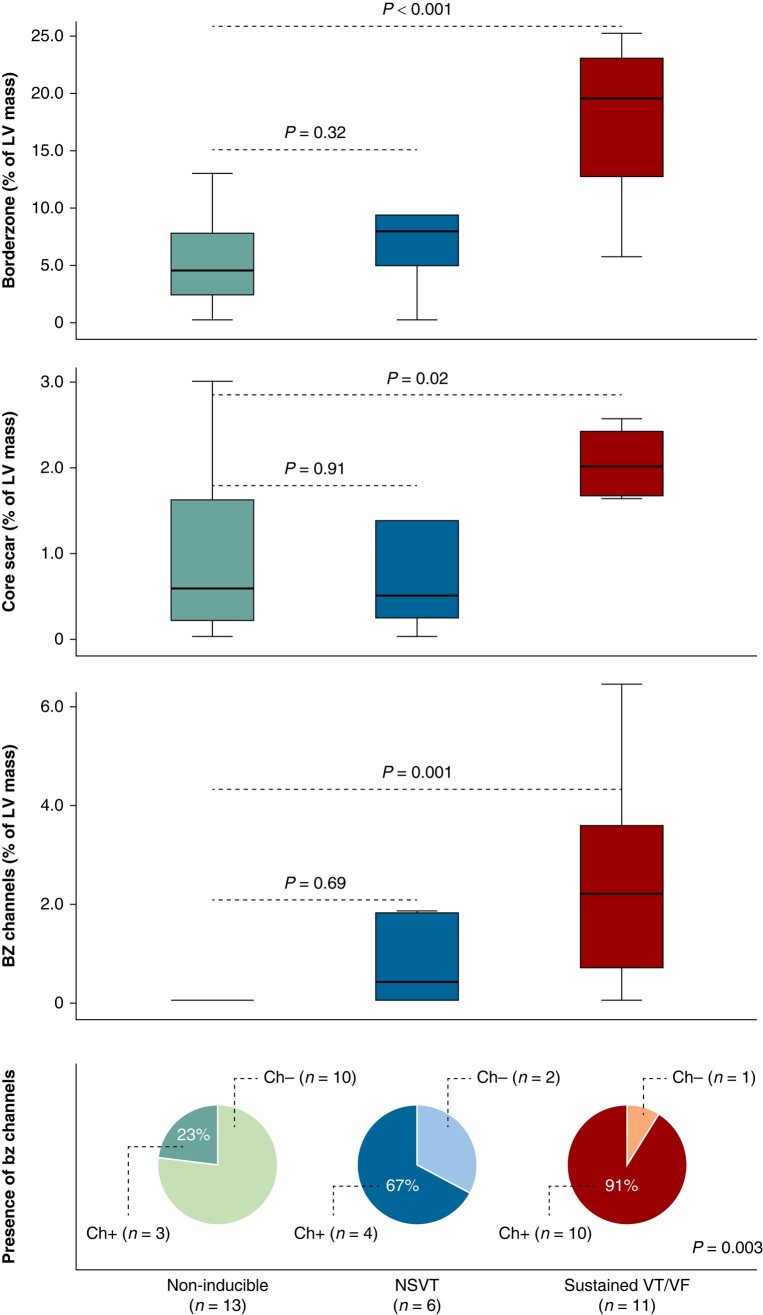
Scar features in inducible and non-inducible HCM patients. Box plots comparing BZ, core and BZ channel mass in patients with non-inducible, inducible non-sustained, and inducible sustained VAs. Pie charts indicate the prevalence of BZ channels in the same patients.

LV mass was comparable in non-inducible (102 g, IQR: 91–176), inducible non-sustained (111 g, IQR: 97–156), and inducible sustained VA patients (106 g, IQR: 99–191) (*P* = 0.82). On the contrary, scar mass showed a progressive increase between groups (5.2%, IQR: 0.8–11.9% vs. 8.2%, IQR: 4.1–14.2 vs. 21.4, IQR: 13.7–27.4; *P* = 0.002) in both its borderzone and dense core components (*Figure 1*). Borderzone channels were found in 3 (23.1%) out of 13 non-inducible, 4 (66.7%) out of 6 inducible non-sustained VA, and 10 (90.9%) out of 11 inducible sustained VA patients (*P* = 0.003). Channel mass markedly differed between groups and was substantially higher in sustained VA patients (*Figure 1*).

**Figure 2:**
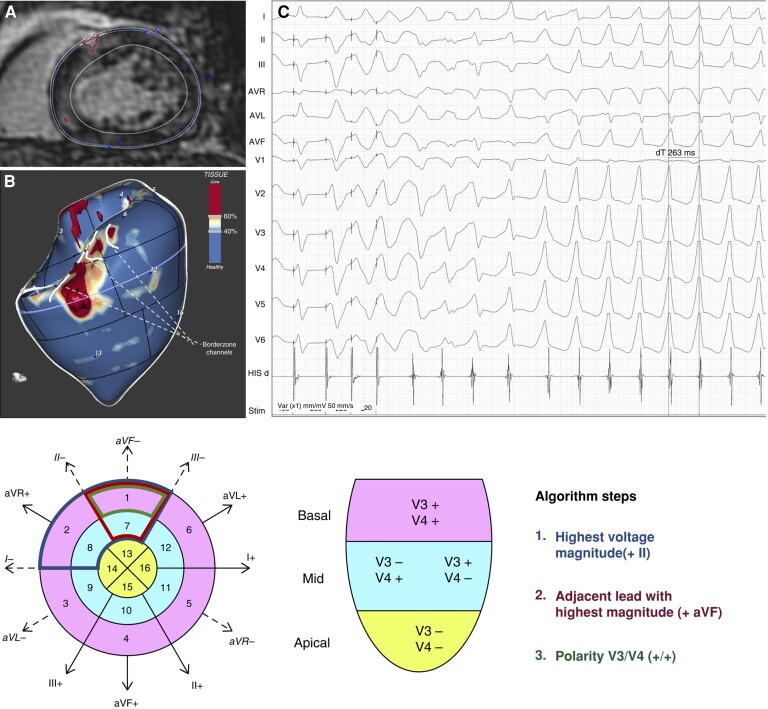
Anterior scar-related monomorphic VT and QRS axis-based algorithm. (*A*) Cardiac magnetic resonance short axis view showing a patchy spot of anterior and septal LGE. (*B*) Left ventricular 3D model with colour-coded tissues according to signal intensity. Scar dense core is red, BZ orange and white, and healthy myocardium is blue. A BZ channel is drawn over the surface as a white line, extending between normal myocardium zones. The upper and lower boundaries of the depicted channel are protected from the mitral annulus and a core scar area, respectively. The BZ channel is located at the level of the LV Segment 1 (basal anterior). (*C*) A PVS-induced VT is shown. The VT initiates as a short run of PVT and then organizes into a monomorphic sustained rapid-rate VT (CL: 262 ms). In agreement with scar channel location, the 12-lead ECG shows concordant positive QRS complexes from V2–V6 and frontal plane axis compatible with origin from the anterior segment of the LV base. (*D*) Application of the QRS axis-based algorithm. The process involves two steps. First, the limb lead with the highest voltage magnitude (II) is identified. The adjacent lead with the highest magnitude (aVF) determines the group of segments that are suggested as a potential site of origin. Secondly, concordant positive (++) precordial leads V3 and V4 are identified, suggesting origin from Segment 1.^18^

**Figure 3:**
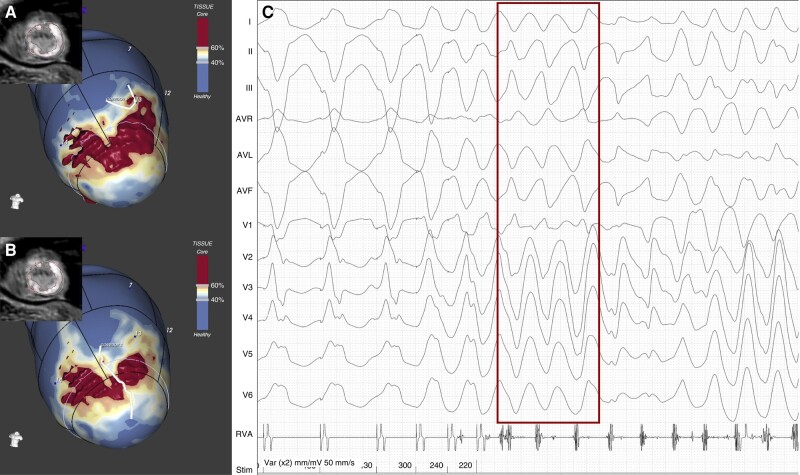
Apical scar-related monomorphic-initiated PVT. Three-dimensional 80% (*A*) and 90% (*B*) LV myocardial shells are visualized, representing the most epicardial strata of the LV. Scar dense core is coded in red, BZ in orange and white, and healthy myocardium in blue. Two BZ channels (white lines) are drawn at the level of the 80% and 90% myocardial layers, respectively, extending between normal myocardium zones and being protected from dense scar. Both channels are located at the level of the Segment 13. (*C*) A PVS-induced PVT is shown. Before degenerating into sustained PVT, a short run of MMVT is observed (red frame) with QRS axis and precordial transition compatible with origin from the antero-lateral LV apex, where myocardial scar and BZ channels are located.^18^

**Figure 4:**
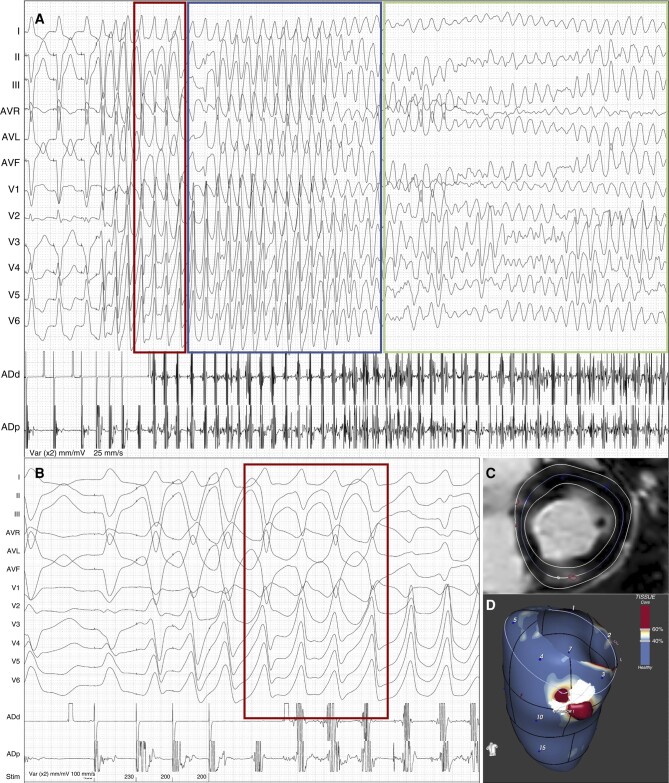
Basal scar-related VT degenerating into VF. (*A*) During PVS using three extrastimuli at the RV apex, a short run of monomorphic VT is induced (red square, beats 1–4) that rapidly degenerates into PVT (blue square) and then VF (green square). (*B*) The short run of MMVT (red frame) shows QRS axis and precordial transition compatible with origin from the basal portion of the inferior LV, where the myocardial scar and BZ channel are located. (*C*) CMR short axis view showing a patchy spot of inferior LGE. (*D*) Three-dimensional 50% LV myocardial layer is visualized, representing a mid-myocardial stratum. Scar dense core is coded in red, BZ in orange and white, and healthy myocardium in blue. A short BZ channel (white line) is drawn extending between normal myocardium zones and being protected from dense scar. The channel is located at the level of the LV Segment 4 (basal inferior).

All 13 patients who exhibited inducibility with sustained or non-sustained MMVT or MI-PVT had myocardial scar. In 10 out of 13 (77%) of these VTs, the LV segment of origin, as determined on 12-lead ECG, corresponded with the location of LGE. Eight out of 10 of these LGE regions presented BZ channels (*Figures 2*–*4*).

### Clinical management and follow-up

Management options were discussed with patients based on clinical risk factors and inducibility of sustained VT/VF at PVS. Six out of 19 patients without inducible sustained VA received an ICD based on baseline risk profile, while all patients with inducible sustained VA were implanted. Conditional VT zone was programmed in 13 patients (76%). VT zone detection rate and intervals were 182 ± 18 bpm and 31 ± 5 beats, respectively. VF zone detection rate was 215 ± 16 bpm with 30/40 intervals in most patients. During a median follow-up of 20 months (IQR: 7–37) after PVS, none of the patients died or had resuscitated cardiac arrest. One patient had an appropriate ICD shock for VF. This patient was a 54 years-old man with family history of SCD as single major risk factor, low (3.4%) 5-year risk of SCD according to the ESC estimator, LV scar with one BZ channel, and inducible sustained PVT at PVS.

## Discussion

In this study, we investigated whether the architecture of myocardial scar, as assessed by LGE-CMR, affects the inducibility of VA and establishes substrates that determine the electrophysiological characteristics of inducible VA in HCM patients. We found a gradual increase in the mass of BZ and dense scar tissue and the prevalence of BZ channels among patients, progressing from those who were non-inducible, to those who were inducible but had non-sustained VA, and finally to those with sustained VA. Additionally, the majority of MMVT or MI-PVT/VF had an ECG origin that aligned with the location of LGE and BZ channels.

Previous studies have shown that in HCM patients the extent of myocardial scarring is associated with SCD^3^ and inducibility of VA at PVS.^14^ Our study adds to this body of evidence by demonstrating that not only the amount of fibrosis but also the composition of the scar is relevant to the inducibility and the characteristics of VA in HCM patients. Indeed, patients in whom BZ channels were identified were more likely to have inducible arrhythmias. This finding is consistent with the notion that regions of viable myocardial tissue within the BZ protected by non-conductive scar tissue can create anatomic isthmuses that serve as slow-conducting re-entrant pathways, which may contribute to the maintenance of VT circuits.^19^ In this view, our study goes beyond previous reports by investigating the relationship between scar architecture and VA, which is a further step towards identifying the substrates entailing VA in HCM. Compared to that previously described in ischaemic cardiomyopathy^9^ and in patients with left ventricular dysfunction,^8^ the average mass of BZ channels identified in this population is notably lower, potentially stemming from the distinct pathophysiologic origin and configuration of scar tissue in these different clinical settings. Indeed, HCM exhibits a distinct pathological profile from ischaemic cardiomyopathy, featuring more diffuse fibrosis caused by hypertrophy and microvascular ischaemia, rather than dense scarring within a vascular territory.^20,21^ Moreover, the mass of BZ channels identified in this population is lower as compared to a distinct HCM cohort,^12^ a difference that could be attributed to the higher scar mass and arrhythmic susceptibility observed within that group of patients, all of whom were high-risk ICD recipients.

Our findings also provide some insight into the pathophysiology of VA in HCM. Polymorphic VT/VF has traditionally been regarded as the most common mode of arrhythmic death and the most frequently induced arrhythmia at PVS in HCM patients.^22,23^ However, its anatomical and functional substrate is unclear. Indeed, most spontaneous non-sustained arrhythmias in HCM are monomorphic, and a substantial proportion of ICD interventions in HCM patients are due to MMVTs.^24–29^ Notably, we observed that PVT/VF induced with PVS is often preceded by a monomorphic VA that originates from within the scar. These MMVTs may use BZ channels as critical components of re-entry circuits and rapidly degenerate into disorganized arrhythmias in the specific context of the HCM myocardium, which is characterized by a disorganized sarcomeric alignment and non-uniform anisotropic conduction, favouring high degree of activation dispersion, slow conduction and unidirectional blocks.^30^ This hypothesis is corroborated by the finding that the location of scar segments comprising protected BZ channels is related to the ECG-derived site of origin of MMVTs, suggesting that these VAs originate from a specific substrate that is localized within the scar.^31^ It is important to emphasize that this study suggests scar-related re-entry as the primary arrhythmogenic mechanism for induced, rather than spontaneous, arrhythmias. Furthermore, this observation is made within a patient population characterized by a high prevalence of LGE. It is known that VA can occur in patients with HCM without structural remodelling or distinct areas of replacement fibrosis. These events are likely promoted by intracellular mechanisms following pathological changes in ion currents and intracellular Ca^2+^ homoeostasis.^32^

As far as risk stratification is concerned, previous studies in historical HCM series have reported variable and conflicting results regarding the value of PVS.^22,33–35^ More recently, it has been suggested that positive PVS predicts spontaneous VA in HCM,^13^ and that inducibility of VA is more common in subjects with a higher mass of myocardial fibrosis.^14^ Our study is of pathophysiologic nature and does not aim, given the small sample size and short follow-up, to draw clinical conclusions on the role of PVS in predicting the risk of VA in HCM patients. Nevertheless, our current findings establish a foundation for the hypothesis that PVS could unveil inducible VA and assist in risk stratification among individuals with complex LGE patterns. This holds particular relevance when traditional risk assessment falls short of providing conclusive criteria for ICD candidacy. Also, they emphasize the importance of a more nuanced approach to the evaluation of LGE in HCM.

### Study limitations

Our study has several limitations. First, it is a single-centre study with a small sample size, which may limit the generalizability of our findings. However, published series of HCM patients that underwent LGE-CMR and PVS are limited. Secondly, the follow-up for arrhythmic events was too short to fully capture the long-term predictive value of our results. Thirdly, the number of patients without LGE in this study is too small to evaluate the significance of VA inducibility in these subjects. Fourthly, in this retrospective study we didn’t include patients who refused PVS after shared decision-making on ICD implantation using the available ESC or AHA/ACC risk models. Therefore, there is a bias in our selection that precludes drawing conclusions about the prognostic role of PVS in unselected HCM patients. Finally, while we identified BZ channels using LGE-CMR post-processing, we did not perform invasive mapping to confirm their role as slow-conducting myocardial corridors actively involved in sustaining re-entry and VTs, which warrants further investigation.

## Conclusions

Scar architecture affects the inducibility and determines the electrophysiological characteristics of inducible VA in HCM patients. A high proportion of induced VA originates as MMVT at the segments were scar and BZ channels are present. If confirmed in larger series, these findings could assist risk stratification, especially in patients with complex LGE patterns in which the decision to implant an ICD needs to be individualized.

## Data Availability

The experimental data used to support the findings of this study are available from the corresponding author upon reasonable request.
